# Low awareness of risk mitigation prescribing in response to dual crises of COVID-19 and overdose deaths among people who use unregulated drugs in Vancouver, Canada

**DOI:** 10.1186/s12954-022-00632-6

**Published:** 2022-05-25

**Authors:** Mana Moshkforoush, Kora DeBeck, Rupinder Brar, Nadia Fairbairn, Zishan Cui, M.-J. Milloy, Jane Buxton, Tanis Oldenburger, Will McLellan, Perry Kendall, Kali Sedgemore, Dean Wilson, Thomas Kerr, Kanna Hayashi

**Affiliations:** 1 BC Centre on Substance Use, Vancouver, Canada; 2 School of Public Policy, Simon Fraser University, Burnaby, Canada; 3 Regional Addiction Program, Vancouver Coastal Health, Vancouver, Canada; 4 Department of Medicine, University of British Columbia, Vancouver, Canada; 5 BC Centre for Disease Control, Vancouver, Canada; 6 Mountainside Harm Reduction Society, Chilliwack, Canada; 7 Faculty of Health Sciences, Simon Fraser University, Burnaby, Canada

**Keywords:** Drug overdose, Safe supply, Harm reduction, COVID-19

## Abstract

**Background:**

When the novel coronavirus pandemic emerged in March 2020, many settings across Canada and the USA were already contending with an existing crisis of drug overdoses due to the toxic unregulated drug supply. In response, the Canadian province of British Columbia (BC) released innovative risk mitigation prescribing (RMP) guidelines for medical professionals to prescribe pharmaceutical alternatives to unregulated drugs in an effort to support the self-isolation of people who use unregulated drugs (PWUD) in preventing both SARS-CoV-2 virus infection and overdoses. We sought to assess the level of awareness of RMP and identify factors associated with this awareness among PWUD in Vancouver, BC.

**Methods:**

Cross-sectional data were derived from participants enrolled in three community-recruited prospective cohort studies of PWUD in Vancouver, interviewed between July and November 2020. Multivariable logistic regression analysis was used to identify factors associated with awareness of RMP.

**Results:**

Among 633 participants, 302 (47.7%) had heard of RMP. Of those 302 participants, 199 (65.9%) had never tried to access RMP services, ten (3.3%) made an unsuccessful attempt to access RMP, and 93 (30.8%) received RMP. In the multivariable analysis, participants who had awareness of RMP guidelines were more likely to self-identify as white (adjusted odds ratio [AOR] = 1.47; 95% confidence interval [CI]: 1.01, 2.13), to have completed secondary school education or higher (AOR = 1.67; 95% CI: 1.16, 2.39), to have used drugs at a supervised consumption or overdose prevention site in the past six months (AOR = 1.66; 95% CI: 1.10, 2.52), and to have received opioid agonist therapy as treatment for opioid use disorder in the past six months (AOR = 1.51; 95% CI: 1.02, 2.24).

**Conclusion:**

At least four months after the release of the guidelines, RMP was known to less than half of our study participants, warranting urgent educational efforts for PWUD, particularly among racialized groups and those who were not accessing other harm reduction services. Furthermore, the majority of participants who were aware of RMP guidelines had never tried to access the service, suggesting the need to improve perceived accessibility and knowledge of eligibility criteria.

## Background

The USA and Canada have been contenting with a public health crisis of overdose deaths attributed to synthetic opioids such as fentanyl and its analogues. Fentanyl has a rapid onset [1], is estimated to be up to 100 times more potent than morphine, and its lethal doses are often small when compared to other opioids [2]. In the USA, almost 50,000 people died from overdose deaths involving opioids in 2019, more than double the number in 2010 [3]. In Canada, 26,690 deaths were attributable to opioid toxicity between January 2016 and September 2021, with Canada’s western provinces experiencing the highest population-adjusted rates [4].

On April 14, 2016, the province of BC declared a public health emergency due to escalating opioid overdoses and related deaths [5]. Since then, the BC Coroners Service reports that unregulated drugs have claimed the lives of more than 9,000 British Columbians [6]. The year 2021 saw the highest loss of life at 2,232 reported deaths, following a 127% increase in the rate of death due to unregulated drug toxicity since 2019. The drastic rise in the overdose crisis is largely attributable to the compounding effects of the novel coronavirus disease (COVID-19) pandemic, which was declared a public health emergency in BC on March 17, 2020. Prior to March 2020, the number of deaths per month had remained in the double digits for almost one year [6]. Since the onset of the pandemic, the dual crises of COVID-19 and opioid overdose have seen over 100 deaths per month. Notably, BC recorded 210 and 215 deaths in November and December 2021, respectively. These are the two largest numbers of suspected deaths ever recorded in a month in the province’s history and equate to about 7 deaths per day [7].

The effects of the COVID-19 pandemic on the overdose crisis were not unexpected. PWUD are at heightened risk of SARS-CoV-2 virus infection due to high rates of related comorbidities and exposure to conditions where following physical distancing and personal hygiene recommendations are challenging or impossible [8–10]. Moreover, public health measures in response to the COVID-19 crisis were expected to pose significant challenges to existing mitigation efforts in the overdose crisis. The redistribution of healthcare resources to COVID-19 containment risked decreasing the availability of direct healthcare services for PWUD, such as community health centers and outreach services. Physical distancing measures risked interrupting access to key overdose prevention services, such as substance use disorder treatments (e.g., opioid agonist therapy [OAT]), as well as supervised consumption and overdose prevention sites. Additionally, previous reports documented that COVID-19-related social and policy changes affected the unregulated drug supply in North America in several ways. As borders closed, the availability and potency of certain drugs decreased, prices increased or stayed the same for more diluted drugs, and cheaper and more profitable fentanyl that is easily trafficked via mail increased the toxicity of the unregulated drug supply [11, 12]. These fluctuations in drug availability often led PWUD to substitute for other substances or use inconsistently, which would in turn affect their tolerance levels [13]. In response to concerns and evidence of a worsening overdose crisis in tandem with an emerging COVID-19 crisis, the province of BC launched dual risk (i.e., overdose and COVID-19) mitigation services for PWUD.

Risk mitigation prescribing (RMP) guidelines [14], also known as pandemic prescribing or “(prescribed) safer supply” (though the terminology has evolved since), were released in March 2020 and allowed physicians and nurse practitioners to prescribe certain pharmaceuticals (e.g., short-acting opioids such as hydromorphone) to people who use unregulated drugs, with the costs covered by the province’s universal no-cost healthcare system. Under the guidelines, a patient prescribed OAT (e.g., those prescribed methadone) who continues to use unregulated opioids can also be prescribed short-acting opioids (on top of their long-acting OAT medications), often for take-home dosing. By providing pharmaceutical alternatives to the toxic unregulated drug supply, the interventions were intended to 1) prevent the spread of COVID-19 by enabling physical distancing and self-isolation among PWUD, and 2) prevent overdose deaths by reducing the consumption of toxic street drugs. Eligibility criteria for RMP included: being an at-risk, suspected or confirmed case of COVID-19 infection; having history of ongoing active substance use (opioids, stimulants, alcohol, benzodiazepines or tobacco); and having high risk of withdrawal, overdose, craving or other harms related to drug use.

Despite its important objectives, implementation of RMP has been limited. There are no previously established approaches to prescribing for overdose prevention versus addiction treatment, and regulatory bodies have been slow to provide guidance on how physicians may interpret RMP guidelines in lieu of the traditional practices surrounding the prescription of opioids and concerns of diversion [15]. The uptake of RMP in BC has also been highly centralized to the Vancouver Coastal Health region that covers urban Vancouver [16], with the highest rates of prescribing expected in the city’s Downtown Eastside (DTES) neighborhood. This is an area characterized by high rates of poverty, homelessness, mental illness, violence and marginalization, and is known to be the epicenter of substance use-related harms in the province of BC [17–20]. Our study is focused on this geographic region since the early impacts of RMP were most likely to be observed here. The uptake of RMP is directly dependent on the awareness of these services among PWUD. Therefore, the objective of our study was to assess the level of awareness of RMP and to identify factors associated with this awareness among PWUD in Vancouver, BC, during the first eight months of RMP implementation in 2020.

## Methods

Data were drawn from three ongoing prospective cohort studies of PWUD in Vancouver: the Vancouver Injection Drug Users Study (VIDUS), the AIDS Care Cohort to evaluate Exposure to Survival Services (ACCESS), and the At-Risk Youth Study (ARYS). Further details of these cohorts are available elsewhere [21–23]. In brief, VIDUS enrolls HIV-seronegative adults (≥ 18 years) who injected drugs in the month prior to enrollment. ACCESS enrolls HIV-seropositive adults who used an unregulated drug other than or in addition to cannabis in the month prior to enrollment. ARYS enrolls street-involved youth aged 14 to 26 years who used an unregulated drug other than or in addition to cannabis in the month prior to enrollment. The studies use harmonized data collection and follow-up procedures to allow for merged data analyses. All three cohorts administer identical questionnaires by trained interviewers and serologic tests for HIV and hepatitis C virus at equal follow-up frequency (i.e., every six months). Upon completion of each biannual study visit, participants receive a $40 CAD honorarium. All three studies have received ethics approval from the University of British Columbia/Providence Health Care Research Ethics Board.

Due to the coinciding onset of the COVID-19 pandemic, the questionnaire used in this study was administered remotely via phone between July and November 2020. We included in our analyses all participants who completed the questionnaire and who reported using unregulated drugs in the six months prior to the interview date, excluding those who exclusively used cannabis. The questionnaire contained COVID-19 specific measures, including measures related to RMP. In the present analyses, the main outcome of interest was a binary measure of awareness of RMP guidelines, defined as having ever “heard about the safer drug supply guidelines” (yes vs. no). Before asking this question, interviewers read the following statement to the participants: “In March 2020, new provincial guidelines were released in order to allow access to prescription drugs for some people who use drugs during the COVID-19 public health emergency. The guidelines are often called safer drug supply guidelines.”

Covariates were selected based on hypothesized relationships with the awareness of RMP guidelines according to Rhodes’ Risk Environment framework [24], which prompted us to consider a range of social and structural factors in addition to individual factors. Specifically, the explanatory variables of interest included the following sociodemographic characteristics: age (per year increase, continuous); self-identified ethnicity/ancestry (white vs. Black, Indigenous or other people of color [BIPOC]); self-identified gender (male vs. female or other gender minorities); education (≥ secondary school education vs. < secondary school education); and residence in the DTES neighborhood of Vancouver. COVID-19 related variables included having ever been tested for COVID-19, ever accessed COVID-19 emergency housing options by the government, and inability to self-isolate or social distance. Variables related to drug-use included the following measures: ≥ daily use of alcohol; ≥ daily use of unregulated opioids (including heroin, unregulated fentanyl or *down*); ≥ daily use of stimulants (including cocaine, crack or crystal methamphetamine); use of benzodiazepines; and having experienced a non-fatal overdose. Additional variables of interest included the following social/structural exposures: involvement in drug dealing; incarceration; use of drugs at supervised consumption or overdose prevention sites; engagement in addiction treatment (OAT [any of buprenorphine-naloxone, methadone, long-acting oral morphine, injectable opioids] vs. non-OAT addiction treatment only [e.g., detox, residential treatment] vs. none); engagement in treatment for mental health; and reporting inability to access health or social services. We also included cohort designation (ACCESS vs. ARYS vs. VIDUS). All variables except for cohort, age, ethnicity/ancestry, self-identified gender, education, COVID-19 testing, and access to housing options by the government referred to the six months prior to the interview date.

Bivariable and multivariable logistic regression analyses were used to identify factors associated with awareness of RMP guidelines. All covariates of interest were included in the multivariable model, and collinearity was assessed by the variance inflation factor (VIF) with a cutoff of 5.0 [25]. In sub-analyses, we examined descriptive statistics of participants’ access to RMP (able to access vs. unable to access vs. never tried to access) among those who were aware of RMP guidelines. All statistical tests were two-sided and considered statistically significant at *p* < 0.05. All analyses were conducted using SAS version 9.4 [26].

## Results

In total, 884 participants completed interviews during the study period. Of those, 242 participants (101 VIDUS, 68 ACCESS, and 73 ARYS participants) did not use drugs or exclusively used cannabis in the six months prior to the interview date, and nine participants did not answer the main outcome question. These 251 participants were excluded from the analysis. Among the analytic sample composed of the remaining 633 participants, 342 (57.5%) were male; 336 (56.8%) were white, 231 (39.0%) were Indigenous, and 29 (4.9%) were Black or other people of color; 309 (48.8%) resided in the DTES in the past six months; and the median age was 45 (1st and 3rd quartile: 32, 55) years.

As shown in Table 1, 302 (47.7%) participants had heard of RMP guidelines. The level of RMP awareness was almost identical among those who had ever been tested for COVID-19 (47.3%) to that of the entire sample. In total, three (0.5%) participants reported having ever tested positive for COVID-19. The results of the multivariable analysis showed that participants who had awareness of RMP guidelines were more likely to self-identify as white (adjusted odds ratio [AOR] = 1.47; 95% confidence interval [CI]: 1.01, 2.13), to have completed secondary school education or higher (AOR = 1.67; 95% CI: 1.16, 2.39), to have used drugs at a supervised consumption or overdose prevention site (AOR = 1.66; 95% CI: 1.10, 2.52), and to have received OAT as addiction treatment (AOR = 1.51; 95% CI: 1.02, 2.24). Collinearity was not detected using VIF (all < 2.0).

**Table 1:** Bivariable and multivariable logistic regression analysis of factors associated with awareness of RMP (*n* = 633)

	Heard of risk mitigation prescribing (*n*, %)			
Variable	Yes (302, 47.7%)	No (331, 52.3%)	OR [95% CI]	AOR [95% CI]
**Cohort**				
ACCESS	90 (29.8%)	111 (33.5%)	1.13 [0.90, 1.41]	1.12 [0.74, 1.70]
ARYS	92 (30.5%)	92 (27.8%)	0.91 [0.73, 1.15]	1.41 [0.72, 2.76]
VIDUS	120 (39.7%)	128 (38.7%)	Reference	Reference
**Age**, per year increase (median, 1^st^ – 3^rd^ quartile)	42 (31–54)	45 (33–55)	0.99 [0.98, 1.00]	0.99 [0.97, 1.01]
**White** (vs. Black, Indigenous, other people of color)	166 (60.1%)	166 (52.5%)	1.36 [0.98, 1.89]	**1.47 [1.01, 2.13]**
**Male** (vs. female and other gender minorities)	155 (55.8%)	187 (59.0%)	0.88 [0.63, 1.21]	0.91 [0.63, 1.33]
** ≥ Secondary school education** (vs. < ss education)	146 (53.7%)	134 (42.7%)	**1.56 [1.12, 2.16]**	**1.67 [1.16, 2.39]**
**Residency in DTES** (vs. in other neighborhoods)^a^	157 (52.0%)	152 (45.9%)	1.28 [0.93, 1.74]	1.33 [0.90, 1.96]
**Tested for COVID-19** (vs. not tested)	87 (28.8%)	97 (29.6%)	0.96 [0.68, 1.36]	0.99 [0.68, 1.44]
**Access to housing options by the government** (vs. no access/unsure of access)	7 (2.3%)	8 (2.4%)	0.96 [0.35, 2.69]	0.96 [0.26, 3.60]
**Unable to self-isolate or social distance** (vs. able)	80 (26.9%)	97 (29.9%)	0.87 [0.61, 1.23]	0.73 [0.48, 1.09]
**≥ Daily alcohol use** (vs. < daily use)^a^	33 (11.0%)	51 (15.6%)	0.67 [0.42, 1.07]	0.75 [0.45, 1.26]
**≥ Daily unregulated opioid use** (vs. < daily use)^a,b^	150 (49.7%)	136 (41.1%)	**1.41 [1.03, 1.94]**	1.09 [0.73, 1.63]
**≥ Daily stimulant use** (vs. < daily use)^a,b^	134 (44.4%)	115 (34.7%)	**1.50 [1.09, 2.06]**	1.29 [0.88, 1.88]
**Benzo use** (vs. no benzo use)^a,b^	18 (6.0%)	11 (3.3%)	1.84 [0.86, 3.97]	1.82 [0.77, 4.32]
**Use of drugs at supervised consumption site** (vs. no drug use at supervised consumption site)^a^	97 (32.1%)	75 (22.7%)	**1.62 [1.13, 2.30]**	**1.66 [1.10, 2.52]**
**Non-fatal overdose** (vs. no overdose)^a^	47 (15.7%)	55 (16.8%)	0.92 [0.60, 1.41]	0.85 [0.53, 1.36]
**Incarceration** (vs. no incarceration)^a^	10 (3.3%)	20 (6.1%)	0.53 [0.25, 1.16]	0.51 [0.20, 1.30]
**Involvement in drug dealing** (vs. no involvement)^a^	105 (34.8%)	89 (26.9%)	**1.45 [1.03, 2.03]**	1.19 [0.80, 1.75]
Addiction treatment^a^				
OAT	184 (61.1%)	171 (52.3%)	**1.52 [1.08, 2.12]**	**1.51 [1.02, 2.24]**
Non-OAT treatment only	24 (8.0%)	25 (7.7%)	1.35 [0.73, 2.51]	1.19 [0.59, 2.42]
No treatment	93 (30.9%)	131 (40.1%)	Reference	Reference
**Unable to access health or social services** (vs. able to access/never tried to access)^a^	82 (27.4%)	86 (26.0%)	1.08 [0.76, 1.53]	1.08 [0.72, 1.62]
**Treatment for mental health** (vs. no treatment)^a^	87 (29.0%)	72 (21.9%)	**1.46 [1.02, 2.09]**	1.36 [0.91, 2.03]

OR: odds ratio. AOR: adjusted odds ratio. CI: confidence interval. DTES: Downtown Eastside neighborhood of Vancouver. OAT: opioid agonist therapy

(A)ORs in bold font denote *p* < 0.05

^a^Denotes behaviors/events in the past 6 months

^b^Denotes drug use via injection or non-injection

**Fig. 1:**
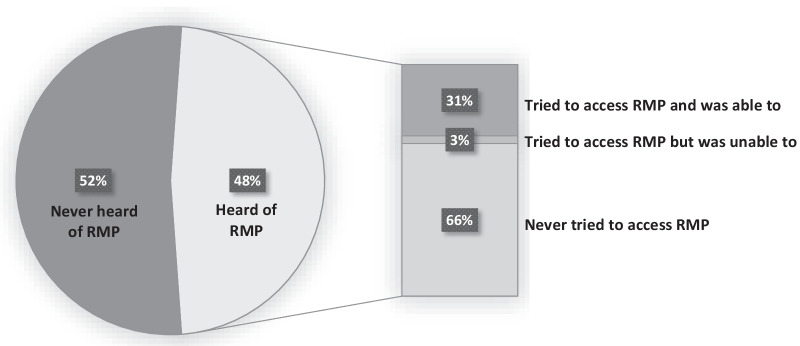
Awareness of RMP among participants who never tried to access RMP

In the sub-analysis, as shown in Fig. 1, among 302 participants who were aware of RMP guidelines, 199 (65.9%) had never tried to access RMP services, 10 (3.3%) had tried to access RMP but were unable to receive it, and 93 (30.8%) received RMP.

## Discussion

To our knowledge, this is the first study to quantify the level of awareness of RMP guidelines and identify factors associated with this awareness among a community-recruited sample of PWUD. In our study, less than half of participants (47.7%) had awareness of RMP at least four months after the release of the guidelines. In the multivariable analysis, participants who self-identified as white, had completed secondary school education or higher, used drugs at a supervised consumption or overdose prevention site in the past six months, or received OAT as addiction treatment in the past six months were more likely to have heard of RMP guidelines. Of further concern, two-thirds of those who were aware of RMP had never tried to access the intervention.

BC’s Vancouver Coastal Health region, in particular Vancouver’s DTES neighborhood, has seen the highest number of prescriptions to date [16]. Our study sampled directly from this target population (55.4% of participants resided in the DTES) yet still displayed low awareness of RMP within the epicenter of the overdose crisis in the province. This further demonstrates that awareness of RMP is low and suggests that knowledge dissemination may be key to the implementation and uptake of this service. The low level of awareness of RMP identified in our study may be attributed to a few factors. First, input from the Regional Addictions Program at Vancouver Coastal Health suggests that the intervention was primarily characterized as COVID-19 prevention (i.e., to support self-isolation) for those who are at risk of overdose, rather than overdose prevention alone. This characterization may have rendered RMP less relevant to our study population. While our study instrument did not allow us to measure the full scale of the need for self-isolation (e.g., either the participant themselves or someone residing with them testing positive for COVID-19), only 29.2% had ever been tested for COVID-19. Only 0.5% of the study sample reported testing positive for COVID-19, compared to 16.3% who reported experiencing an accidental overdose in the past six months. Among participants who were perceived to be at risk of COVID-19 and were tested for infection, awareness of RMP was still low at 47.3%. This further demonstrates that a lack of strategic advertising of RMP as an overdose prevention measure may have negatively impacted RMP roll out.

Our findings indicate that participants who had awareness of RMP were more likely to have used drugs at a supervised consumption or overdose prevention site. These sites have the added function of providing opportunities to learn about other harm reduction and treatment services (e.g., RMP) from peer workers – PWUD trained in overdose response [27]—and of facilitating peer social networks and community connections among PWUD [28]. Previous literature has shown the importance of these connections and of word-of-mouth knowledge dissemination for public health education [29]. Namely, in a study of fentanyl risk knowledge among PWUD in Vancouver, BC, a significant majority of participants who were aware of risks associated with fentanyl reported learning about them through word-of-mouth [29]. From the findings in our study, we speculate that the disruption of peer social networks and natural modes of knowledge dissemination due to COVID-19 may also have partially contributed to the low level of awareness of RMP among the population of PWUD. During the pandemic, peers were discouraged from having guests where they were housed in supportive housing units, and from socializing at overdose prevention sites. These measures impacted the ability for peer networks to gather in-person and engage in education and advocacy on the same level that they were achieving in years prior to the pandemic. Beyond PWUD-to-PWUD knowledge dissemination—through peers in the community, or peer workers at supervised consumption or overdose prevention sites—outreach workers in community settings also play an important role in knowledge dissemination, raising awareness of RMP, and health literacy in general. While our study did not collect data on PWUD’s contact with outreach workers, we speculate that the healthcare system’s shift in focus from community/preventative care to acute care and infection control during the pandemic also hampered community outreach efforts.

Our study also found an association between RMP awareness and having received OAT in the six months prior to the interview date. Similar to the opportunities for learning about RMP at supervised consumption or overdose prevention sites, having an OAT prescriber likely provides opportunities to learn about other services and speak with a potential prescriber of RMP. In fact, the vast majority of prescribers of RMP are physicians who are OAT prescribers. A patient may or may not be on OAT at the time of their visit with a physician, but physicians are likely to be in a position to offer eligible patients a spectrum of options from OAT to RMP, or both. While the association between OAT and awareness of other interventions such as RMP reinforces the benefits of OAT for those eligible, it also reveals the shortcomings of a medicalized model of “safer supply” as many PWUD may not be connected to healthcare providers and may not have an opioid use disorder diagnosis, but still be at risk of overdose (the main target of RMP). Furthermore, many PWUD who are not OAT recipients already may be less likely to engage with health services such as OAT clinics due to past experiences of stigma, institutionalized racism, etc.

Participants who self-identify as white (vs. BIPOC) were more likely to have heard of RMP guidelines. As there are multiple sources to obtain knowledge about RMP (e.g., through peer networks, primary care workers, etc.), the present study cannot determine which sources were most likely associated with the observed racial disparities in the knowledge of RMP. While future research needs to investigate this issue in more depth, one previous study of racism in healthcare settings makes note of the “double whammy” effect, whereby Indigenous PWUD in Vancouver perceive acts of discrimination as a result of being both visibly Indigenous and residing in the DTES [30]. These acts of discrimination often manifest as clinical practice that is negatively influenced by stigmatizing racial stereotypes. Indigenous participants in the above study reported being reluctant to disclose their substance use due to the fear of receiving inadequate medical care as a result, and notably lacked awareness of services devoted to Indigenous health. This lack of awareness of targeted services parallels our findings of low awareness of RMP among non-white PWUD. The fear of stigmatizing racial stereotypes may have also created a barrier for BIPOC, and in particular Indigenous, participants to talk to prescribers about RMP. However, additional research is required to unpack this issue.

Finally, participants who completed secondary school education or higher were more likely to have heard of RMP guidelines. This finding is consistent with previous literature, in which a predictive model of health literacy found that educational attainment was the strongest predictor of health literacy [31]. If health literacy is defined as “the ability to find, understand, and use information and services to inform health-related decisions” [32], then low awareness of RMP among a population of PWUD in Vancouver may be considered an indicator of low health literacy. Considering this link between educational attainment and health literacy, we suggest that higher educational attainment—and an associated higher health literacy—among our study population would support higher awareness of risk mitigation services such as RMP. Since overdose prevention is an urgent crisis, a quicker solution to addressing the low awareness among those with low educational attainment is to use terminology that is clear, comprehensible and free from medical or legal jargon. Information relevant to PWUD should be targeted to PWUD, rather than to prescribers and health officials alone. Unfortunately, the wording, length and complexity of the RMP guidelines likely contributed to the low awareness among individuals with lower educational attainment in our cohort.

While increasing awareness of RMP is the first step in facilitating implementation of the guidelines, we also found that the majority of those who were aware of RMP had never tried to access it. This is concerning as it suggests that PWUD not only lack sufficient awareness of RMP, but also that those who do have awareness are facing barriers to accessing the service. RMP is somewhat unique in that it is a public health intervention being provided in an individualized fashion and at the discretion of prescribers. This is different from other public health interventions, such as naloxone, where PWUD do not require a prescription to access the intervention. This dependence on prescribers creates a number of barriers, including physician willingness, comfort and awareness to prescribe RMP, as well as requiring patient engagement with the health system. The roll out of RMP generally lacked public awareness campaigns, and the onus was on PWUD to seek information and find a potential prescriber. For RMP to be successful as a public health intervention, similar approaches as made for other public health interventions would be needed, such as broad availability without prescribers as gatekeepers and funding to implement education campaigns that target the population meant to benefit from the intervention. Further implications of our findings include the need to improve perceived accessibility and knowledge of eligibility criteria, especially since a plan for expanding RMP was introduced on July 15, 2021 [33]. With policy directives that focus on expansion of eligibility, medications and coverage, awareness of RMP and other derivative programs are crucial to achieving the overarching goal of preventing overdose deaths. Once awareness is achieved, additional research is needed to explore and address why the majority of PWUD who had awareness of RMP did not try to access the service.

This study has several limitations. First, all measures were self-reported, which may have introduced response bias into our results. However, self-reported measures among PWUD have been shown to be generally reliable and valid [34–36]. Second, the cross-sectional nature of this study does not allow us to address the temporal sequence of associations found in our study. Lastly, recruitment of the cohort participants was conducted through nonprobability sampling methods rather than random sampling, and data collection through phone interviews may have introduced selection bias. These study design issues may reduce the external validity of our results such that the level of awareness of RMP found in our study cannot be generalized to all PWUD in Vancouver.

## Conclusions

Despite the intended purpose of mitigating urgent risks of overdose and spread of COVID-19, only about half of our sample of PWUD in Vancouver had heard of RMP at least four months after the release of the guidelines. Participants who had awareness of RMP guidelines were more likely to self-identify as white, to have completed secondary school education or higher, to have used drugs at a supervised consumption or overdose prevention site, and to have received OAT as addiction treatment. Of further concern, among study participants who were aware of RMP guidelines, roughly two-thirds had never tried to access RMP. As RMP is currently being expanded as a more permanent overdose prevention intervention, these findings suggest the need to increase the awareness of RMP guidelines, particularly among racialized people and those who are not connected with key harm reduction services. Additional research is also needed to explore and address the reasons for low uptake of RMP among individuals who have heard of the intervention.

## Data Availability

The data used for this study are not publicly available and can be requested from the corresponding author on reasonable request and with permission of the University of British Columbia/Providence Health Care Research Ethics Board.

## Abbreviations

ACCESS: AIDS Care Cohort to evaluate Exposure to Survival Services
AOR: Adjusted odds ratio
ARYS: At-Risk Youth Study
BC: British Columbia
BIPOC: Black, Indigenous, or other people of color
CI: Confidence interval
DTES: Downtown Eastside
OAT: Opioid agonist therapy
PWUD: People who use drugs
RMP: Risk mitigation prescribing
VIDUS: Vancouver Injection Drug Users Study
VIF: Variance inflation factor
